# The Structural Design and Optimization of a Novel Independently Driven Bionic Ornithopter

**DOI:** 10.3390/biomimetics10060401

**Published:** 2025-06-13

**Authors:** Mouhui Dai, Ruien Wu, Mingxuan Ye, Kai Gao, Bin Chen, Xinwang Tao, Zhijie Fan

**Affiliations:** 1 College of Mechanical and Vehicle Engineering, Changsha University of Science & Technology, Changsha 410114, China; 202203130528@stu.csust.edu (M.D.); 202103130816@stu.csust.edu (M.Y.); cbzr520@csust.edu.cn (B.C.); fzj18707310852@163.com (Z.F.); 2 College of International Engineering, Changsha University of Science & Technology, Changsha 410114, China; 202227010204@stu.csust.edu.cn (R.W.); 202327050320@stu.csust.edu.cn (X.T.); 3 Hunan Key Laboratory of Smart Roadway and Cooperative Vehicle-Infrastructure Systems, Changsha 410114, China

**Keywords:** bionic ornithopter, independently driven mechanism, dual-motor drive, structural optimization, aerodynamic performance, flight stability, stress analysis, simulation validation

## Abstract

To address the limitations of traditional single-motor bionic ornithopters in terms of environmental adaptability and lift capacity, this study proposes a dual-motor independently driven system utilizing a cross-shaft single-gear crank mechanism to achieve adjustable flap speed and wing frequency, thereby enabling asymmetric flapping for enhanced environmental adaptability. The design integrates a two-stage reduction gear group to optimize torque transmission and an S1223 high-lift airfoil to improve aerodynamic efficiency. Multiphysics simulations combining computational fluid dynamics (CFD) and finite element analysis (FEA) demonstrate that, under flapping frequencies of 1–3.45 Hz and wind speeds of 1.2–3 m/s, the optimized model achieves 50% and 60% improvements in lift and thrust coefficients, respectively, compared to the baseline. Concurrently, peak stress in critical components (e.g., cam disks and wing rods) is reduced by 37% to 41 MPa, with significantly improved stress uniformity. These results validate the dual-motor system’s capability to dynamically adapt to turbulent airflow through the precise control of wing kinematics, offering innovative solutions for applications such as aerial inspection and precision agriculture.

## 1. Introduction

A bionic ornithopter is a novel type of bionic robot, which can simulate the movement of biological wings. According to the principle of biological flight, the bionic ornithopter has a wide range of applications in the field of national defense and in civilian fields, and it has excellent characteristics such as being small and flexible, being good at concealment, and having a high aerodynamic efficiency. For instance, Ye et al. proposed a multi-plane method combined with quasi-steady blade element theory to analyze the aerodynamics of hovering flapping wings, highlighting the impact of wing deformation on aerodynamic forces and efficiency, which offers new theoretical insights for ornithopter design [1]. The bionic ornithopter has garnered significant attention from researchers in recent years and has made notable research advancements. These are two representative examples. The Smart Bird, produced by German Pneumatic Company Festo in 2011, is a bionic ornithopter resembling a seagull-like aircraft; its total weight is 450 g, the body is made of carbon fiber and composite materials, and the wings spread out to about 2 m. It is equipped with two rotating wheels and adopts a four-link compound mechanism with symmetrical distribution, which can realize the flapping-wing movement and the twisting movement of the wing tip at the same time [2] to provide power for flight. The four-bar linkage mechanism, as a fundamental component in many ornithopters, has been extensively studied for its ability to mimic the complex wing motion of birds. Recent research by Garg et al. [3] further demonstrated the effectiveness of such mechanisms in achieving stable and efficient flight, highlighting their potential to enhance flight performance. This provides valuable insights into the design of advanced ornithopter systems.

In China, many universities and research institutions are studying bionic ornithopters. The bionic ornithopter Dove [4] developed by Professor Song Bifeng’s research team at Northwestern Polytechnical University is the leading example. The birds we observe are very complex and changeable in flight and can react quickly to protect themselves in extreme conditions. When encountering strong winds, birds will change the flap frequency of their wings in time, so that the lift on both sides of the wings is the same, and they can ensure stable flight. Recent research by Mu et al. [5] explored the aerodynamic characteristics of two types of foldable flapping-wing mechanisms. Their findings demonstrated that an asymmetric flapping mode of foldable wing structures can significantly enhance aerodynamic performance. This discovery aligns with the principles of our dual-motor independently driven system, which allows for the precise control of wing kinematics to optimize lift and thrust. Therefore, when we study the bionic ornithopter, we should learn more about the flight principle of birds themselves, rather than simply studying it as an ordinary aircraft. Only in this way can we design a better bionic ornithopter.

The ongoing research and development of bionic ornithopters have brought them closer to reality. However, current models are predominantly driven by a single motor, resulting in a symmetrical wing flap at the same frequency. While this design reduces overall weight and ensures stable lift with its simplicity, it also limits its ability to fly in adverse weather conditions. Li et al. showed that introducing torsional motion (2 DOF) to a bird-like flapping wing could significantly boost the lift and thrust coefficients by increasing pressure differentials and intensifying vortex structures [6]. Strong winds can cause a significant imbalance between the left and right sides, rendering the tail wing ineffective and leading to a potential loss of altitude.

Recent advancements in plasma actuator technology have shown promise for enhancing aerodynamic control without adding significant weight. Patel et al. demonstrated that dielectric barrier discharge (DBD) air actuators can effectively manipulate airflow over highly swept wings, providing control authority at high angles of attack without mechanical moving parts. Similarly, Neretti et al. [7] conducted extensive experiments on DBD plasma actuators, highlighting their potential for active flow control applications. These findings suggest that plasma actuators could offer a lightweight alternative for improving the maneuverability and stability of ornithopters in complex airflow conditions. Moayedi and Mohaddes Deylami showed that attaching a Gurney flap and DBD plasma actuator to an NACA 0012 airfoil improves the lift coefficient and aerodynamic performance by increasing pressure differentials and optimizing vortex structures [8].

The bionic ornithopter designed in this paper differs from traditional ornithopters. The independently driven bionic ornithopter utilizes two motors to power both wings. In the face of strong winds, the flapping frequency of both wings can be adjusted by controlling and modifying the lift force to enhance the flight capability of the ornithopter. Additionally, the independent drive system can provide greater thrust to both wings, improving the flight-carrying capacity and enabling high-altitude operations. Furthermore, it can also extend the service life of parts and motors, unlike traditional ornithopters with a single motor drive that must overcome resistance from both wings, leading to stress concentration and potential damage to transmission parts such as cranks and rockers. As Geißler [9] highlighted, single-motor systems face challenges in maintaining stable flight under turbulent conditions due to their reliance on complex torque compensation mechanisms. These mechanisms not only increase structural complexity and weight but also limit adaptability to adverse weather conditions. In contrast, the dual-motor independently driven system offers a more efficient solution by distributing mechanical loads evenly, thereby enhancing both flight performance and component durability. The independent drive system evenly distributes resistance and external forces between two motors, reducing stress on transmission parts and enhancing their service life. The study by Johansson also emphasized the importance of adjusting wingbeat kinematics to optimize the thrust-to-lift ratio, which aligns with the principles of the dual-motor system employed in our ornithopter. By enabling independent control of each wing’s motion, our design can dynamically adjust the thrust and lift forces, much like how birds fine-tune their wing movements to achieve optimal aerodynamic efficiency [10].

This paper focuses on the structural design and optimization of a novel independently driven bionic ornithopter. By employing a dual-motor drive system and an S1223 high-lift airfoil, the study aims to enhance the ornithopter’s environmental adaptability and flight stability. Multiphysics simulations combining computational fluid dynamics (CFD) and finite element analysis (FEA) demonstrate significant improvements in the lift and thrust coefficients, as well as stress uniformity in critical components. These advancements pave the way for practical applications in aerial inspection and precision agriculture, where adaptability and stability are paramount.

## 2. Common Bionic Ornithopter Transmission Structure

### 2.1. The Single-Gear Crank–Rocker Mechanism

**Figure 1:**
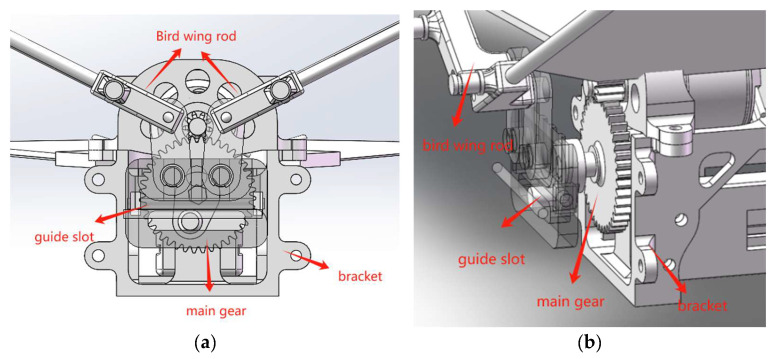
The 3D model of a single-gear crank–rocker mechanism: (**a**) the front of the model; (**b**) the side of the model.

As illustrated in Figure 1, the single-gear crank–rocker mechanism is a straightforward transmission configuration characterized by its simplicity. This structure, which drives two wings to flap through a gear, contrasts with more intricate mechanical designs. Its simplicity and lightweight characteristics make it an attractive option in many conditions. However, this mechanism is unsuitable for high-speed rotation due to the inertia and centrifugal forces generated by the rocker, which can lead to vibration and imbalance at higher speeds. Moreover, the crank experiences increased resistance, resulting in expansion and contraction at high frequency for extended periods. This can potentially cause damage to the parts and hinder long-term efficient operation.

### 2.2. The Double-Gear Crank–Rocker Mechanism

The double-gear crank–rocker mechanism is characterized by a high degree of symmetry, with two large gears independently controlling the rocker for each wing. Upon activation, the motor propels two identical large gears through the reduction gear group, which in turn drives the rocker motion, ultimately facilitating wing flap. Throughout the movement process, the friction between gear groups remains relatively low. As a result, a high-ratio gear can be designed to operate here. This is beneficial for reducing the weight of the engine and increasing the final output torque [11].

However, this structure has several significant drawbacks. Firstly, the manufacturing precision of the double-gear crank–rocker mechanism is stringent, leading to challenging gear processing and assembly, and a relatively elevated manufacturing cost. Secondly, the compact nature of the double-gear crank–rocker structure necessitates additional support points when designing the frame, thereby escalating the complexity of frame design. In addition, the presence of gear play and slippage within the mechanism creates friction as the crank moves and energy is lost during the transmission process, resulting in a significant reduction in the transmission efficiency of the double-gear crank–rocker mechanism.

### 2.3. The Cross-Shaft Single-Gear Crank Mechanism

**Figure 2:**
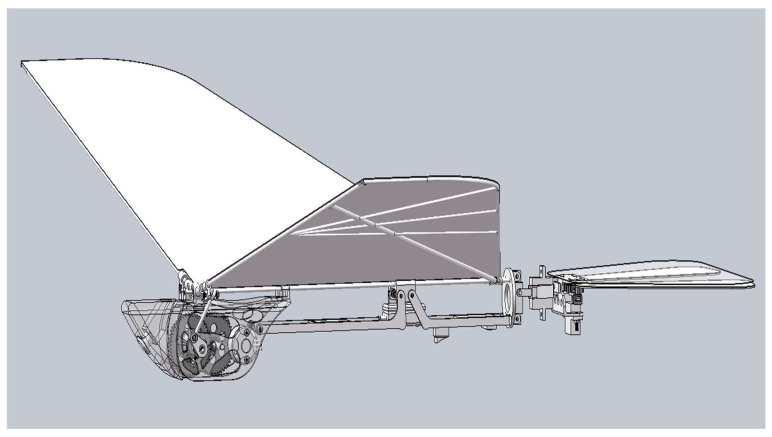
The 3D model of a cross-shaft single-gear crank mechanism.

The cross-shaft single-gear crank mechanism, as shown in Figure 2, is a variant of the double-gear crank–rocker mechanism. By integrating the skeleton and limiting mechanisms, the number of components is significantly reduced, leading to an increase in space utilization and cost savings. Moreover, this mechanism ensures high transmission efficiency and stability during rotation. In comparison to the double-gear crank–rocker structure, the cross-shaft single-gear crank design diminishes the size of the ornithopter’s head, preventing it from becoming overly bloated. However, this structure only uses one motor, which limits the ornithopter’s load capacity, making it unable to fly with excessive weights.

After summarizing and analyzing the existing ornithopter transmission mechanisms, it was determined that the cross-shaft single-gear crank mechanism has the most potential for flight applications. In this study, a bionic ornithopter with an independent drive system is designed utilizing the cross-shaft single-gear crank mechanism, thereby enhancing the load capacity of the ornithopter through a dual-motor drive.

## 3. A Novel Type of Independently Driven Bionic Ornithopter

**Figure 3:**
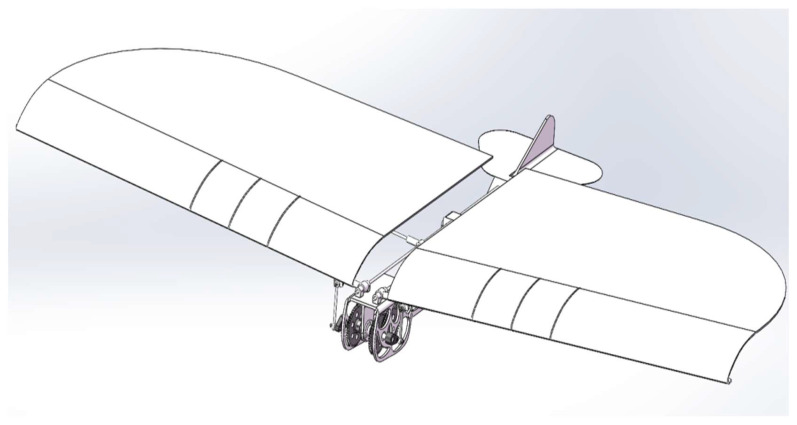
The 3D model of a novel type of independently driven bionic ornithopter.

In order to overcome the above-mentioned disadvantages of ornithopters, a novel type of independently driven ornithopter structure is designed, as illustrated in Figure 3. This structure incorporates two motors and two sets of gears, that independently drive each side of the wings, ensuring stability during flight. Compared to traditional transmission structures, this design has the following advantages: independent control of both wings, increased lift capacity, strong carrying capacity, and minimal energy loss.

### 3.1. A Pair of Independently Driven Wings

The traditional mechanical structure uses a motor to drive two wings to flap, and the drive mechanism must be symmetrically configured. This ensures that the wings on both sides flap at the same frequency during flight, generating equal thrust and lift forces, and the steering needs to be achieved through the tail-turning fins. However, it is only used in low-wind environments. In the event of severe weather, such as strong winds, the unbalanced lift from the left and right wings can cause the tail control system to become blocked, resulting in a loss of control and a potential crash.

**Figure 4:**
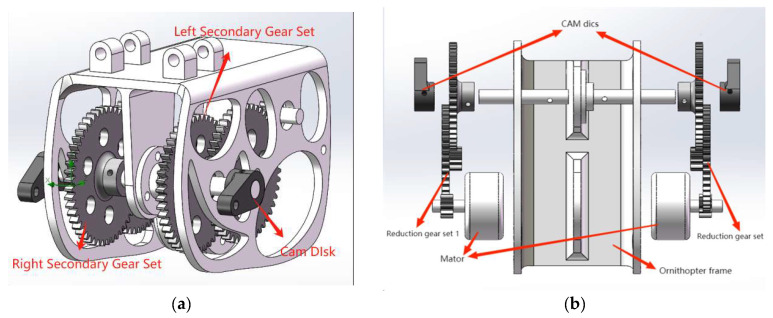
(**a**) The novel type of independently driven bionic ornithopter transmission mechanism. (**b**) An explosion diagram of the transmission structure.

The novel independent drive mechanism enhances the maneuverability and stability of the bionic ornithopter by adding a motor and gear set to the cross-shaft single-gear crank mechanism, as illustrated in Figure 4. This symmetrically mounted mechanism balances the weight of the frame and prevents tilting during flight. The independent driving of both wings is achieved through a two-motor drive design, allowing for flexible and precise control of the flap frequency. This enables smooth flight in good environmental conditions and stable flight in adverse conditions through motor speed adjustments. Additionally, the use of a differential steering system provides greater stability and sensitivity compared to traditional tail steering systems.

**Figure 5:**
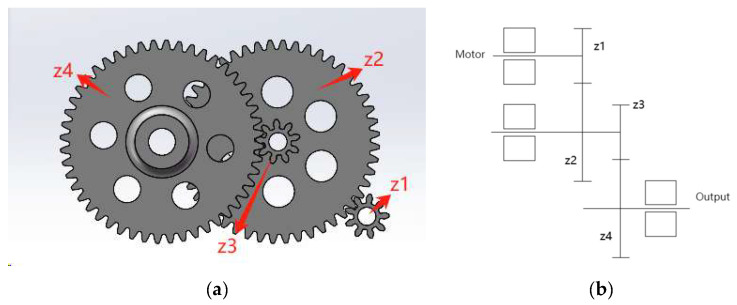
(**a**) A 3D model of the single-sided transmission structure. (**b**) A schematic diagram of the transmission structure.

The 3D model of the single-side gearing is shown in Figure 5. The transmission mechanism consists of a two-stage reduction gear group, which ensures a high transmission efficiency of more than 90% under normal operating conditions. When the ornithopter flaps its wings, it requires a large torque, and the two-stage reduction gear group effectively distributes the load through multistage transmission to provide stable and reliable torque for the ornithopter. Additionally, the compact structure and small volume of the two-stage reducer gear significantly improves space utilization within the ornithopter.

### 3.2. The Choice of Wing Airfoil

**Figure 6:**
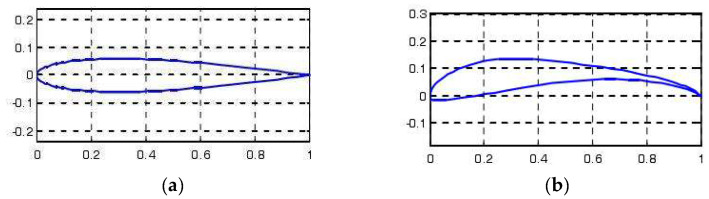
Two common airfoils are depicted. (**a**) The NACA0012 airfoil. (**b**) The S1223 airfoil.

To enhance air mobility, a streamlined airfoil is utilized in the inner section of the wing. Common airfoils are categorized into low-speed, subsonic, and supersonic airfoils. Based on their shapes, they can be divided into flat and concave airfoils as well as S-shaped airfoils [12], such as the two common airfoils shown in Figure 6. Different airfoils have specific applications based on aerodynamic principles; when the pressure on the upper side of the wing is lower than the pressure on the underside, it generates upward lift for improved flight performance and stability. Recent research by Miyasaka et al. [13] has shown that controlling wing motion to modulate aerodynamic force direction is crucial for stable flight in bio-inspired designs, especially under turbulent conditions. This underscores the importance of selecting an airfoil that maximizes lift and minimizes drag to achieve efficient flight performance.

**Figure 7:**
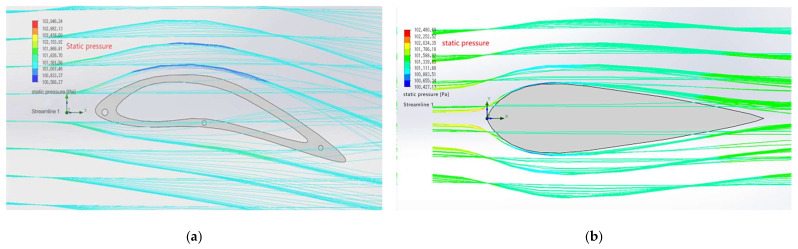
(**a**) Fluid dynamics simulation diagrams of S1223 airfoil. (**b**) Fluid dynamics simulation diagrams of NACA0012 airfoil.

By performing an aerodynamic analysis in Solid Works, we can compare the pressure differences between the upper and lower sides of two airfoils at the same external air flow rate. According to the results shown in Figure 7, under the same conditions, the average pressure on the upper wing surface of the S1223 is about 100,569 Pa, the average pressure on the lower wing surface is about 101,625 Pa, and the pressure difference between the two is about 1056 Pa. However, the upper wing pressure of the NACA0012 is about 100,655 Pa [14], the lower wing pressure is about 100,883 Pa, and the pressure difference between the upper and lower wings is only 218 Pa, which is far less than the pressure difference of the S1223 wing. These differences in pressure coefficients are significant and align with the known aerodynamic performance characteristics of these airfoils. The S1223 airfoil, with its higher lift and lower drag ratio, reduces resistance during ornithopter flight, thus enhancing its overall performance and flap efficiency [15].

Previous research by Park and Yoon demonstrated that non-steady aerodynamic effects, such as delayed stall and wake capture, are crucial for enhancing the flight performance of small ornithopters. Their work highlighted the importance of wing structure and elasticity in optimizing lift and efficiency, which aligns with the principles of selecting an airfoil that maximizes these effects.

Considering characteristics of a flapping-wing flight flow field, it is observed that S1223 has significant curvature and leading edge thickness resembling those found in birds’ inner wings, making it more suitable for flapping-wing flight flow field characteristics [16]. Additionally, S1223 airfoils also demonstrate higher lift and a lower drag ratio, which reduces resistance during ornithopter flight thus enhancing its overall performance and flap efficiency. In general, it is better for ornithopter designers to choose the S1223 wing [17].

**Figure 8:**
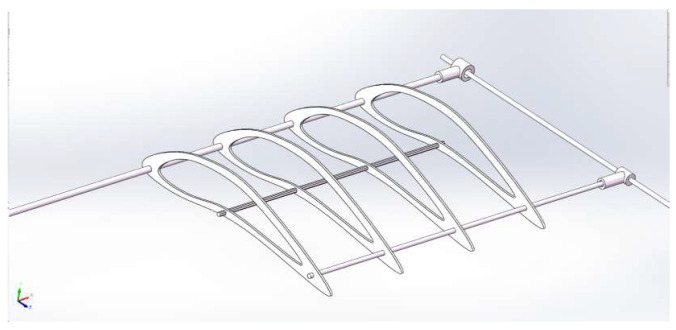
The 3D model of airfoils.

The design of the hollow interior of the wing aims to reduce its weight and ensure that it is not affected by weight during flight. In addition, streamlined wings help to increase lift and thrust, thereby significantly improving the aerodynamic performance of flapping-wing aircraft. The internal wing has four S1223 streamlined wings, which are evenly arranged and connected at their midpoint by square rods, effectively preventing wing rotation relative to the rods and enhancing stability. The mechanism model of the inner wing is shown in Figure 8.

## 4. Strength Verification and Feasibility Analysis

### 4.1. Simulation Analysis of Frame Drop Test

**Figure 9:**
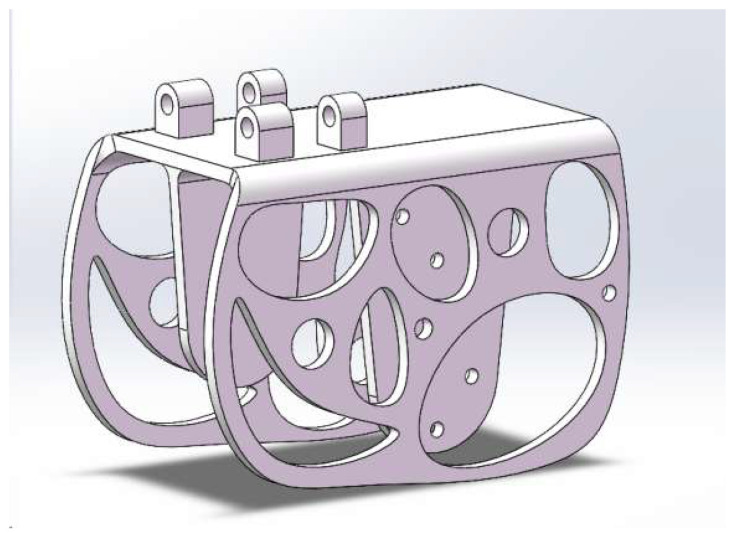
The 3D model of the ornithopter frame.

The entire frame incorporates the integration of a flapping-wing aircraft skeleton and limiting mechanism, as depicted in Figure 9, resulting in a significant reduction in the number of parts and consequently the overall weight. Since a bionic ornithopter cannot land as stably as a fixed-wing UAV, it is easy to generating substantial impact forces during falls and collisions. Therefore, it is imperative to conduct simulations to verify its feasibility.

During actual landing processes, the ornithopter may fall from various angles, with its frame bearing the brunt of an impact upon collision with the ground, potentially leading to deformation. By conducting simulation tests using Solid Works software, we assume that the entire structure falls from a height of 2 m.

*v*^2^ = 2*a* × *h* (1)

By taking the acceleration due to gravity (*a*) as 9.81 m/s^2^, we can calculate an approximate velocity (*v*) of 6.26 m/s—which represents the normal falling velocity.

**Figure 10:**
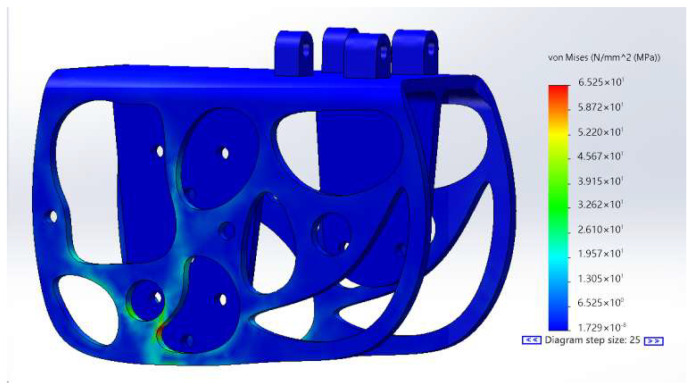
Stress cloud diagram of the ornithopter rack.

Through the Solid Works simulation analysis shown in Figure 10, it is clear that when the frame is dropped from a height of 2 m, the maximum stress reaches about 65.2 MPa, while the average stress is about 10 MPa—both within the acceptable range of the bearing capacity of the components and materials; thus confirming the rationality of the structure.

### 4.2. Gear Design and Strength Verification

The flapping-wing mechanism, which enables the movement of multiple degrees of freedom through a single-drive device, necessitates the implementation of a rational deceleration mechanism in the transmission system to facilitate mechanism action, given the high speed but low torque of the micromotor. The gear reduction mechanism, aside from exhibiting characteristics of stable transmission and high efficiency, is compact and easy to install, facilitating lightweight design. The relationship between the gear transmission ratio, motor speed, and flapping frequency is

*n* = 60*f* × *i* (2)

In Equation (2), *i* represents the gear transmission ratio, *n* represents the brushless motor speed, and *f* represents flapping frequency.

The unloaded rotation speed of the brushless motor is 12,000 r/min, and the oscillation frequency per second is determined to be 7 times in conjunction with the wing area. The reduction ratio calculated by Equation (2) is approximately 28.5, allowing for the design of a two-stage reducer. The modulus of the gear is set at 0.9, with the number of teeth in the large gear being 48 and the number of teeth in the pinion being 9.All parameters of the transmission structure are shown in Table 1.

The pinion material is 40 Cr (tempered), with a tooth surface hardness of 280 HBS; the large gear material is 45 steel (tempered), with a tooth surface hardness of 240 HBS. The number of teeth in the pinion is *Z*1 = 9, the number of teeth in the large gear is *Z*2 = 48, and the gear ratio u = 5.33, assuming a gear operation of 15 years (300 days per year). The motor’s unloaded power is 60 w. (In the following formula, the subscript 1 represents the pinion, and the subscript 2 represents the large gear.)

The torque transmitted (*T*) by the transmission shaft can be obtained by

*T* = 9.55 × 10^6^*P*/*n* = 47.75 N·mm (3)

where *P* is the motor’s unloaded power and *n* represents the brushless motor speed.

(1) Verification based on the tooth surface contact fatigue strength

(4) $$d_{1}\geq \sqrt[{3}]{\frac{2K_{F}T\left(u+1\right){\left(Z_{H}Z_{E}Z_{\varepsilon }\right)}^{2}}{\Phi_{d}u{\left[\sigma_{H}\right]}^{2}}}$$

where *d*_1_ is the minimum gear index circle diameter. The high movement speed and stable transmission result in a load coefficient *K* = 1.3, an area coefficient $Z_{H}$ = 2.5, a elastic influence coefficient $Z_{E}$ = 189.8 ${MPa}^{1/2}$, a coincidence coefficient $Z_{\varepsilon }$ = 0.85, and a tooth width coefficient $\phi_{d}$ = 1. The contact fatigue limit of the pinion is $\sigma_{Hlim1}=$ 600 Mpa.The contact fatigue limit of the large gear is $\sigma_{Hlim1}=$ 550 Mpa. The number of stress cycles is

*N* = 60*n* × *j* × *Lh*. (5)

where *J* is the number of meshing times on the same tooth surface for each revolution of the gear, taking *j* = 1; *Lh* is the working life of the gear (in hours). According to Equation (5), it can be calculated that the number of stress cycles for the small gear is *N_1_* = 5.184 × 10^10^, and the number of stress cycles for the large gear is

*N_2_* = *N_1_*/*u* ≈ 9.7 × 10^9^ (6)

The contact fatigue life coefficients for the small and large gears are *K_HN1_* = 0.90 and *K_HN2_* = 0.95. Where the safety coefficient *S* = 1.

(7) $$[\sigma_{H}]=\frac{K_{HN}*\sigma_{Hlim}}{S}$$

According to Equation (7), the result is $\left[\sigma_{H}\right]$_1_ = 540 MPa and $\left[\sigma_{H}\right]$_2_ = 523 MPa. The smaller figure is taken as the allowable contact fatigue stress of the gear pair. By introducing the data into Equation (4), the result is $d_{1}$ ≥ 6.28 mm. The minimum gear index circle diameter designed by the project is 8.1 mm, which meets the requirements of tooth surface contact strength.

(2) Verification based on the bending strength of the gear tooth root

(8) $$m\geq \sqrt[{3}]{\frac{2K_{F}TY_{\varepsilon }Y_{Fa}Y_{Sa}}{\Phi_{d}Z_{1}^{2}[\sigma_{F}]}}$$

In Equation (8), *m* is the gear modulus. Where *K*_F_ = 1.3, *Φd* is the tooth width coefficient *Φd* = 1. $Y_{\varepsilon }$ is the overlap degree calculated for the bending fatigue strength

(9) $$Y_{\varepsilon }=0.25+\frac{0.75}{\varepsilon_{\alpha }}$$

where $\varepsilon_{\alpha }$ = 1.711. According to Equation (9), the result is $Y_{\varepsilon }$ = 0.688.

The tooth shape coefficients are *Y_Fa1_* = 2.60 and *Y_Fa2_* = 2.20; the stress correction coefficients are *Ysa1* = 1.56 and *Ysa2* = 1.75; the root bending fatigue limits are $\sigma_{Flim1}=500\;MPa$ and $\sigma_{Flim2}=380\;MPa$; the bending fatigue life coefficients are *K_FN1_* = 0.85 and *K_FN2_* = 0.88; and safety coefficient is *S* = 1.4.

(10) $$[\sigma_{F}]=\frac{K_{HFN}*\sigma_{Flim}}{S}$$

According to Equation (10), the results are $\left[\sigma_{F}\right]$_1_ = 303.57 MPa and $\left[\sigma_{F}\right]$_2_ = 238.86 MPa. $\frac{Y_{Fa1}Y_{Sa1}}{{\left[\sigma_{F}\right]}_{1}}=0.0133;\frac{Y_{Fa2}Y_{Sa2}}{{\left[\sigma_{F}\right]}_{2}}=0.0161$.

Take the larger values of the two and put them into Equation (8), and the result is *m* ≥ 0.3. So it is safe to take 0.9 as the gear modulus.

By checking the contact fatigue strength of the tooth surface and the bending strength of the tooth root, it is verified that the parameters of the transmission structure meet the conditions and can be used safely.

### 4.3. Position Control

**Figure 11:**
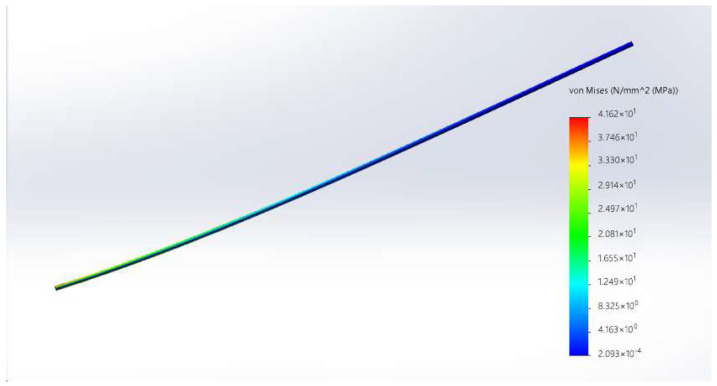
The stress cloud diagram of the wing rod.

During flight, the wing experiences increased resistance and thrust transmitted by the transmission structure. To ensure the wing spar’s load-bearing capacity, carbon fiber was utilized as the material for the wing rod. Static simulation was conducted in Solid Works. The main wing rod body was subjected to a uniform load of 0.001 MPa and an upward force of 10 N. The stress cloud diagram Figure 11 shows that the stress distribution is relatively uniform, with an average stress of about 10MPa and a maximum stress of approximately 41 MPa at the leftmost end. So it is safe to use it.

**Figure 12:**
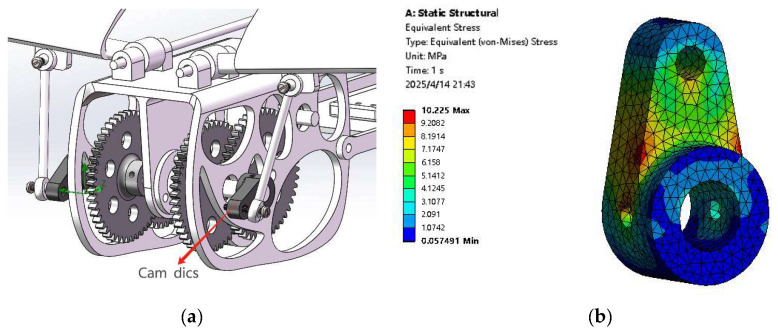
(**a**) The position of the cam disk. (**b**) The stress cloud diagram of the cam disk.

The motor drives the gear group to rotate, transmitting motion to the cam disk through the spindle, which then pushes the stud via the cam disk to achieve a wing flap. Consequently, during rotation, a larger torque is exerted on the cam disk. When the wing flap angle is at its lowest point, during the subsequent rotation process, the cam disk will be subjected to greater reaction force from the stud. In Ansys simulation: provide 1N·m torque to the cam disk and apply a force of 100 N at the joint. As shown in the stress cloud diagram in Figure 12, the maximum equivalent stress reaches 10.225 MPa, and the deformation of the parts is small, thereby extending the service life of the parts.

## 5. Simulation Experiment Data Measurement

### 5.1. Parameter Settings and Mesh Generation

Before carrying out the simulation experiments, it is necessary to set the relevant parameters and perform mesh generation to ensure the accuracy and reliability of the simulation experiments.

#### 5.1.1. Parameter Settings

According to the requirements of the simulation experiment and the actual situation, the following parameters are set:

Flight condition parameters: The flapping frequencies were set to 1 Hz (low frequency), 2 Hz (medium frequency), and 3.45 Hz (high frequency), and the wind speeds were set to 1.2 m/s (low wind speed), 2.5 m/s (medium wind speed), and 3 m/s (high wind speed) to simulate different flight environments, with a flapping amplitude of 30°.

Material parameters: The wing material is carbon-fiber composite, with a density of 1500 kg/m^3^, an elastic modulus of 200 GPa, and a Poisson’s ratio of 0.3. The fuselage material is aluminum alloy, with a density of 2700 kg/m^3^, an elastic modulus of 70 GPa, and a Poisson’s ratio of 0.33.

Simulation time parameters: The simulation time was set to 0.5 s, with a time step of 0.001 s to ensure that the dynamic changes during the flapping process could be captured.

Boundary condition parameters: The inlet wind speed of the fluid domain was set to the above-specified values, and the outlet was set as a pressure outlet with a pressure of 0 Pa. The wing surface was set as a no-slip wall, and the fuselage surface was set as a symmetric boundary.

#### 5.1.2. Mesh Generation

**Figure 13:**
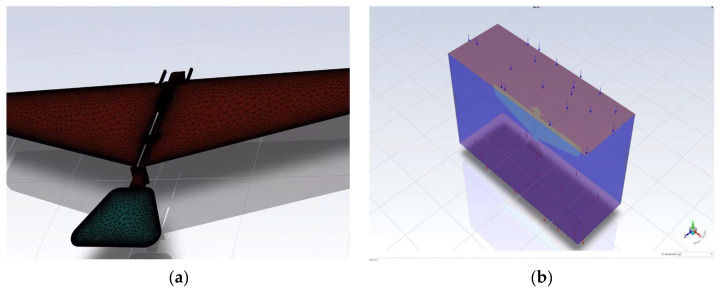
(**a**) The model mesh division diagram. (**b**) The flow field distribution diagram.

Mesh generation was conducted in Gambit, the preprocessor of Fluent. In reality, the flow field of the ornithopter can be considered effectively infinite. However, in computational fluid dynamics (CFD) simulations, it is essential to balance the need for accurate and reliable results with computational performance. Therefore, the computational fluid domain was defined as a rectangular prism. A structured mesh was employed to discretize the model, enhancing both computational accuracy and efficiency. During the mesh generation process, refinement was applied to critical regions such as the wings and fuselage to better capture the details of fluid flow and structural deformation. The total number of mesh elements in the entire model is approximately 500,000, with about 300,000 elements in the wing section and 200,000 elements in the fuselage section. Figure 13 is a schematic diagram illustrating the mesh generation.

### 5.2. Key Algorithms and Data Processing in Fluid Simulation Experiments

#### 5.2.1. Turbulence Model

The standard k-ω SST turbulence model was selected for the aerodynamic numerical simulation in this study. This model is suitable for complex flow characteristics such as low Reynolds numbers, strong unsteadiness, and near-wall shear flows. It can accurately capture boundary-layer development and vortex structure evolution in the near-wall region without wall functions. Additionally, it has a low dependence on grid resolution and demonstrates good convergence and computational stability.

The governing equations for the standard k-ω SST turbulence model are as follows (including the turbulent kinetic energy k equation, the specific dissipation rate equation, and the turbulent viscosity ratio calculation formula, etc.):

$$\frac{\partial (\rho k)}{\partial t}+\frac{\partial (\rho ku_{j})}{\partial x_{j}}=P_{k}-\beta *\rho k\omega +\frac{\partial }{\partial x_{j}}\left[\left(\mu +\sigma_{k}\mu_{t}\right)\frac{\partial k}{\partial x_{j}}\right]$$

$$\frac{\partial (\rho \omega )}{\partial t}+\frac{\partial (\rho \omega u_{j})}{\partial x_{j}}=\alpha \frac{\omega }{k}P_{k}-\beta \rho \omega^{2}+\frac{\partial }{\partial x_{j}}\left[\left(\mu +\sigma_{\omega }\mu_{t}\right)\frac{\partial \omega }{\partial x_{j}}\right]$$

$$\mu_{t}=\frac{\rho k}{\omega }$$

#### 5.2.2. Algorithm Selection

In the numerical simulation of unsteady flow fields, the coupled pressure–velocity coupling format was selected, along with Rhie–Chow interpolation to improve the coupling efficiency and the stability between the pressure and velocity fields. A second-order upwind scheme was used for spatial discretization to ensure numerical stability and effectively capture complex flow features. In addition, Warped-Face Gradient Correction and HighOrderTermRelaxation were activated to further enhance simulation accuracy and iterative convergence.

**Figure 14:**
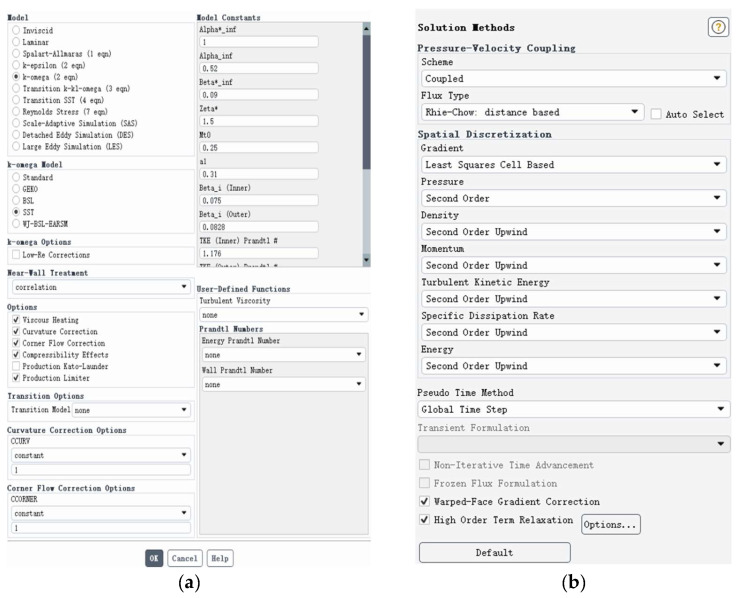
(**a**) Turbulent model selection; (**b**) solver algorithm selection.

The combined application of these solution methods and spatial discretization strategies effectively balances the requirements of high precision, strong coupling, and stability for the numerical simulation of flapping-wing flight, ensuring the reliability and physical consistency of aerodynamic response predictions for flapping wings. When using the CoupledScheme + second-order upwind discretization scheme in Fluent, the core algorithm relies on four key equations. Figure 14 illustrates the selection of the turbulence model and the solver algorithm in this experiment.

$$\left[\begin{array}{cc} A & \nabla (\cdot )\\ \nabla & 0 \end{array}\right]\left[\begin{array}{c} u\\ p \end{array}\right]=\left[\begin{array}{c} b\\ 0 \end{array}\right]$$

$$\phi_{f}=\phi_{C}+\nabla \phi_{C}\cdot r_{f}$$

$$\frac{3\phi^{n+1}-4\phi^{n}+\phi^{n-1}}{2\Delta t}=F(\phi^{n+1})$$

$$\nabla p^{n+1}=\alpha \nabla p^{*}+(1-\alpha )\nabla p^{n}$$

#### 5.2.3. Data Extraction

**Figure 15:**
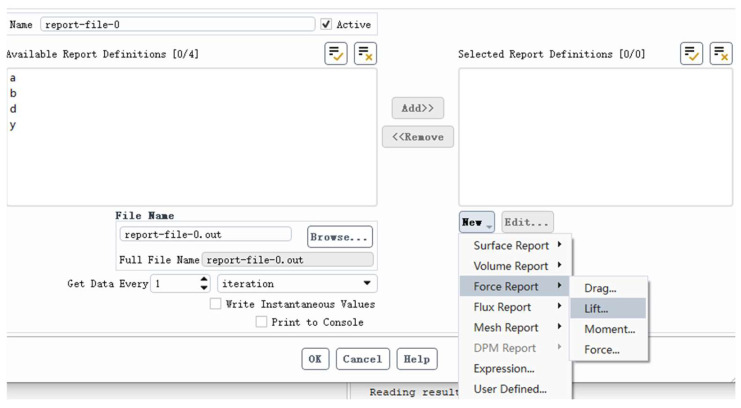
Selection of Flow Field Gas.

Figure 15 presents the workflow for extracting aerodynamic forces. In Fluent, the formulas for calculating lift and drag coefficients are based on the following fundamental equations:

$$C_{L}=\frac{L}{\frac{1}{2}\rho U^{2}A}$$

$$C_{D}=\frac{D}{\frac{1}{2}\rho U^{2}A}$$

As shown in the equation, L denotes lift, D denotes drag,ρ is the fluid density, U represents the free-stream velocity of the fluid, and A is the reference area, typically the projected area of the object.

The steps to extract aerodynamic parameters such as lift and drag in Fluent are as follows:

1. Select “Force Report” or “Surface Report” in the Report Definitions interface.

2. Add the required parameters (e.g., lift and drag) to the “Selected Report Definitions” list via the “Add>>” button.

3. Set the output file name (e.g., report-file = 0.out) and save path.

4. Choose the data acquisition frequency (e.g., per iteration).

5. Check “Write Instantaneous Values” to output instantaneous values or select “Print to Console” to display results directly in the console.

6. Click “OK” to finalize the settings, and Fluent will automatically extract and save the specified aerodynamic data during the computation.

### 5.3. Aerodynamic Force Calculation of Wings

The lift and thrust of the wings were simulated and compared under different conditions (1 Hz-1.2 m/s, 2 Hz-2.5 m/s, and 3.45 Hz-3 m/s).

#### 5.3.1. Lift Data

##### Lift Data Under 1 Hz-1.2 m/s Condition

**Figure 16:**
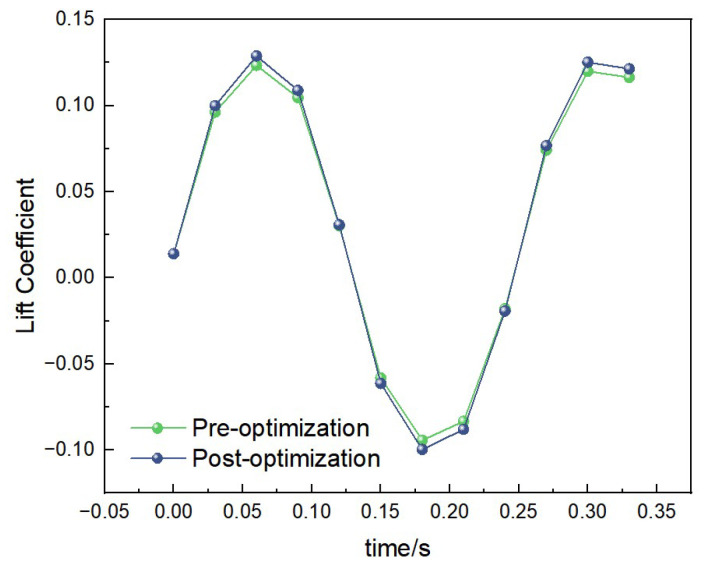
Comparison of lift data under low-frequency and low-wind-speed conditions.

Under the condition of 1 Hz-1.2 m/s, the curves of lift coefficient variation with time for both the pre-optimization and post-optimization models are shown in Figure 16. The lift peak of the post-optimization model further increased, and the variation frequency became more stable. The average lift coefficient of the pre-optimization model was calculated to be 0.08, while that of the post-optimization model was 0.12. This indicates that the post-optimization model has a higher lift performance under low-frequency and low-wind-speed conditions.

##### Lift Data Under 2 Hz-2.5 m/s Condition

**Figure 17:**
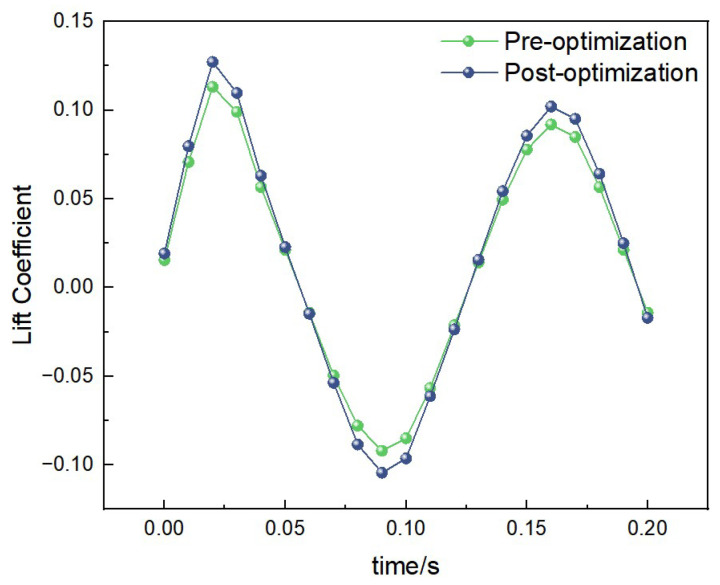
Comparison of lift data under medium-frequency and medium-wind-speed conditions.

Under the condition of 2 Hz-2.5 m/s, the curves of lift coefficient variation with time for both the pre-optimization and post-optimization models are shown in Figure 17. Through calculation, the average lift coefficient of the pre-optimization model is 0.10, while that of the post-optimization model is 0.15. This indicates that the post-optimization model has significantly improved lift performance under medium-frequency and medium-wind-speed conditions.

##### Lift Data Under 3.45 Hz-3 m/s Condition

**Figure 18:**
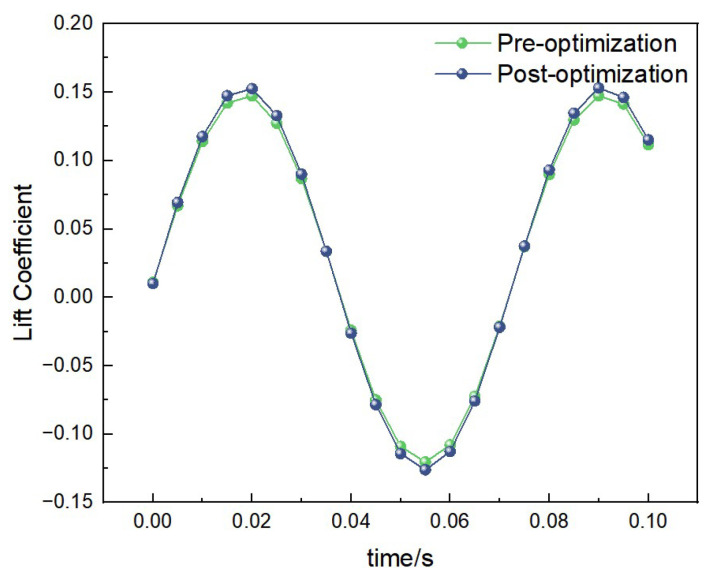
Comparison of lift data under high-frequency and high-wind-speed conditions.

Under the condition of 3.45 Hz-3 m/s, the curves of lift coefficient variation with time for both the pre-optimization and post-optimization models are shown in Figure 18. Through calculation, the average lift coefficient of the pre-optimization model is 0.12, while that of the post-optimization model is 0.18. This indicates that the post-optimization model has stronger lift performance under high-frequency and high-wind-speed conditions.

#### 5.3.2. Thrust Data

##### Thrust Data Under 1 Hz-1.2 m/s Condition

**Figure 19:**
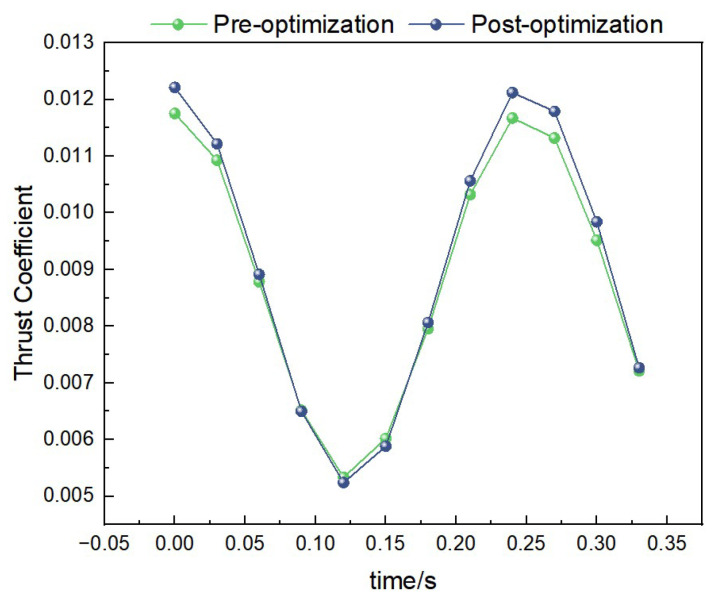
Comparison of thrust data under low-frequency and low-wind-speed conditions.

Under the condition of 1 Hz-1.2 m/s, the curves of thrust coefficient variation with time for both the pre-optimization and post-optimization models are shown in Figure 19. Through calculation, the average thrust coefficient of the pre-optimization model is 0.06, while that of the post-optimization model is 0.10. This indicates that the post-optimization model has higher thrust performance under low-frequency and low-wind-speed conditions.

##### Thrust Data Under 2 Hz-2.5 m/s Condition

**Figure 20:**
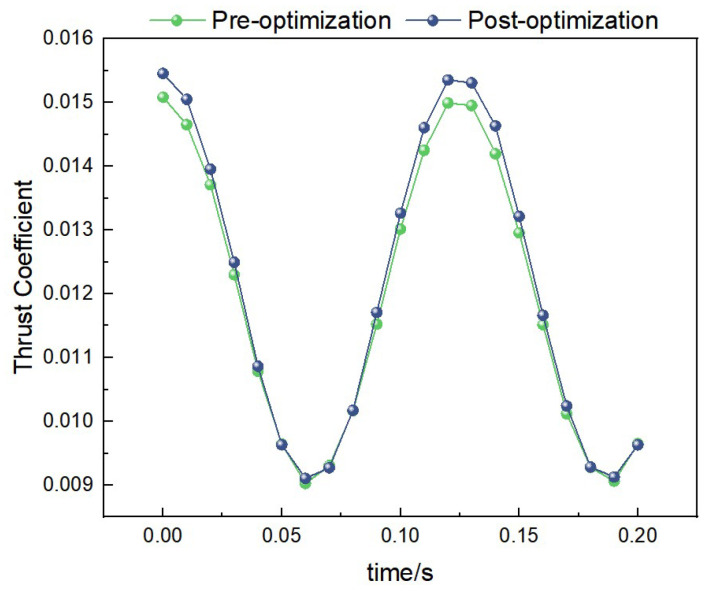
Comparison of thrust data under medium-frequency medium-wind-speed conditions.

Under the condition of 2 Hz-2.5 m/s, the curves of thrust coefficient variation with time for both the pre-optimization and post-optimization models are shown in Figure 20. Through calculation, the average thrust coefficient of the pre-optimization model is 0.08, while that of the post-optimization model is 0.13. This indicates that the post-optimization model has significantly improved thrust performance under medium-frequency and medium-wind-speed conditions.

##### Thrust Data Under 3.45 Hz-3 m/s Condition

**Figure 21:**
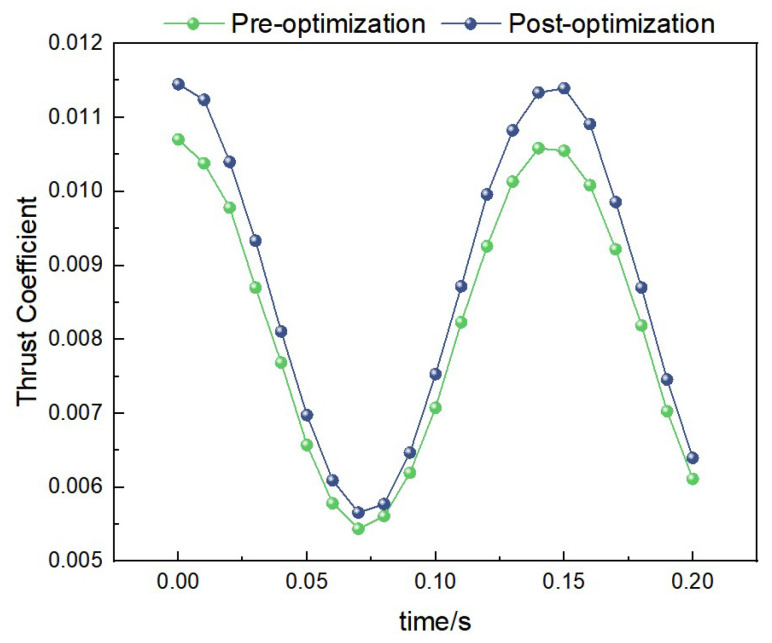
Comparison of thrust data under high-frequency and high-wind-speed conditions.

Under the condition of 3.45 Hz-3 m/s, the curves of thrust coefficient variation with time for both the pre-optimization and post-optimization models are shown in Figure 21. Through calculation, the average thrust coefficient of the pre-optimization model is 0.10, while that of the post-optimization model is 0.16. This indicates that the post-optimization model has stronger thrust performance under high-frequency and high-wind-speed conditions.

#### 5.3.3. Comparative Analysis

By comparing the lift and thrust coefficients of the pre- and post-optimization models under different conditions, the following conclusions can be drawn:

1. Under all frequency and wind speed conditions, the average lift and thrust coefficients of the post-optimization model are significantly higher than those of the pre-optimization model, indicating that the optimized structure has better aerodynamic performance.

2. The lift and thrust coefficients of the post-optimization model show more stable variations under different frequency and wind speed conditions, indicating that the optimized structure has stronger adaptability and stability.

3. The performance improvement of the post-optimization model is particularly significant under high-frequency and high-wind-speed conditions, which is closely related to the optimized independent drive system and dual-motor design, further verifying the effectiveness of the optimization measures.

### 5.4. Stress Cloud Analysis

#### 5.4.1. Stress Distribution Before Optimization

**Figure 22:**
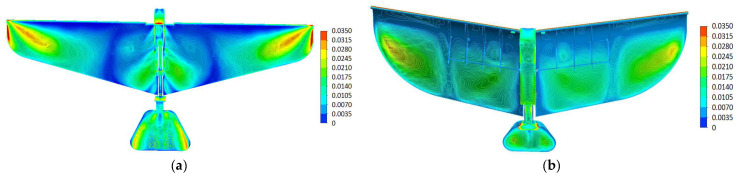
(**a**) The stress distribution diagram of the model before optimization. (**b**) The stress distribution diagram of the model after optimization.

Figure 22a shows the stress distribution of the pre-optimization model. As can be seen from the figure, the stress of the model is mainly concentrated in the leading and trailing edges of the wing, as well as the connection between the wing and the fuselage. The stress values in these areas are relatively high (colors close to red and yellow). The stress in the middle part of the wing is relatively low (colors leaning towards blue and green), indicating that the material utilization rate in these areas is relatively low.

#### 5.4.2. Stress Distribution After Optimization

Figure 22b shows the stress distribution of the post-optimization model. Compared with the pre-optimization model, the stress distribution of the post-optimization model is more uniform. The high-stress areas have been significantly reduced, and the areas originally close to red and yellow have become green or light yellow, indicating that the stress has been effectively dispersed and reduced. The stress values at the leading and trailing edges of the wing and the connection with the fuselage have been significantly reduced. The stress in the middle part of the wing has increased, but it is still within a reasonable range overall (colors leaning towards green and light yellow), indicating that the materials in these areas are used more reasonably, improving the overall structural efficiency.

### 5.5. Comparison with Existing Designs

To better illustrate the advantages of our model, we conducted a detailed comparison with the model analyzed in Lee’s study [18]. In Lee’s research, the ornithopter model exhibited significant stress concentration, with high stress peaks at critical components that could cause premature material failure and reduce structural performance. In contrast, our optimized design has greatly improved this situation. Our model’s wing rod design, with more rational cross-sectional shapes and optimized dimensions, ensures a more even stress distribution and avoids excessive local stress.

**Figure 23:**
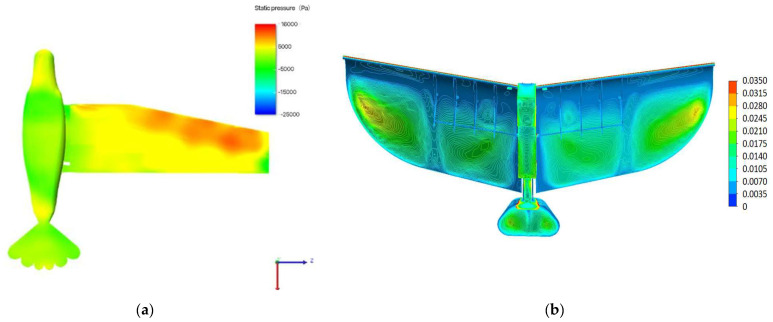
(**a**) The model from Lee’s paper; (**b**) our model featuring even stress distribution and low peak stress.

Figure 23 compares the stress distribution of the two models. On the left is Lee’s model, showing stress concentration areas in red; on the right is our model, with a more uniform stress distribution and lower peak stress.

## 6. Conclusions

An ornithopter based on the principles of bionics is a novel type of flight device that simulates the flight patterns of various aerial creatures to realize flight. It has significant research value in both military and civilian fields due to its excellent characteristics of being small and flexible, having good concealment, and high aerodynamic efficiency. Applications such as power inspections, airport bird deterrence, pesticide spraying, and more, can significantly reduce human resource investments, enhance production efficiency, and facilitate rapid operation. Although the bionic ornithopter has achieved considerable progress to date, there remains a considerable gap between its practical application and its potential, necessitating continuous innovation.

In this study, an independently driven flapping-wing drive mechanism based on the cross-shaft single-gear crank mechanism was designed. This mechanism utilizes two motors to drive each wing separately, enabling each wing to possess distinct flapping frequencies and instantaneous flap speeds. Consequently, the lift force of both wings can be adjusted at any time, thereby endowing ornithopters with the capability to navigate complex airflow. The stability and flexibility of the ornithopter are significantly improved compared to a single-motor drive. Moreover, the dual motor drive enables the sharing of motor load during the flight of the ornithopter, effectively extending the service life of the motors and enhancing the carrying capacity of the ornithopter. The wings of the bionic ornithopter are equipped with S1223 streamlined wings, which facilitate smoother flight by providing greater lift, in accordance with the principles of aerodynamics. Furthermore, the ornithopter’s frame uses an integrated skeleton and limiting mechanism design, which not only reduces the number of components but also facilitates loading, unloading, and maintenance procedures. This design enables regular cleaning, lubrication, and inspection, thereby preventing the failure of and damage to the transmission mechanism.

The simulation experiments conducted provide strong support for the feasibility and superiority of the optimized design. The results show that the optimized ornithopter model has significantly higher lift and thrust coefficients compared to the original model under various frequency and wind speed conditions, demonstrating its superior aerodynamic performance. The stress distribution of the optimized model is also more uniform, indicating better structural performance and reliability. These experimental results fully validate the effectiveness of the optimization measures and highlight the significant performance improvements of the independently driven bionic ornithopter.

In conclusion, the independently driven bionic ornithopter designed in this study represents a novel and optimized structure that outperforms traditional single-motor driven ornithopters in terms of adaptability, stability, and carrying capacity. The simulation experiments have provided solid evidence for its enhanced performance, making it more promising for practical applications. Future work will focus on fabricating the physical model and conducting actual flight tests and aerodynamic experiments to further validate its flight potential and explore its potential in various fields.

## Figures and Tables

**Table 1:** Parameters of transmission structure.

The Name of the Parameter	*i*1		*i*2	
	*Z*1	*Z*2	*Z*3	*Z*4
Gear modulus	0.9			
Number of gear teeth	9	48	9	48
Gear indexing circle diameter/mm	8.1	43.2	8.1	43.2
Transmission ratios at all levels	5.33		5.33	
Total gear ratio	28.4			

## Data Availability

The data presented in this study are available on request from the corresponding author due to privacy.
